# Supporting more affordable and equitable cancer care and research in Lower and Middle Income Countries: *e*cancer’s commitment

**DOI:** 10.3332/ecancer.2021.ed116

**Published:** 2021-11-02

**Authors:** Richard Sullivan, Eduardo Cazap, Danny Burke

**Affiliations:** 1 Institute of Cancer Policy, King’s College London, London, WC2R 2LS, United Kingdom; 2 Editor-in-Chief, ecancer, 13 King Square Avenue, Bristol, BS2 8HU, UK and President, Latin American and Caribbean Society of Medical Oncology (SLACOM), Buenos Aires, Argentina; 3 CEO, ecancer, 13 King Square Avenue, Bristol, BS2 8HU, UK

**Keywords:** Lower and Middle Income Countries (LMICs), open access, education, cancer, patients, accessible, e-learning, videos, events, journal

## Abstract

Oncology professionals in Lower and Middle Income Countries (LMICs) are facing a challenging situation with rising cancer cases together with a lack of educational support and access to relevant research. This article outlines what *e*cancer are doing as an organisation to try to address these issues by providing high quality education in multiple formats for free and supporting authors and readers from LMICs to access and publish research.

## Article

The World Bank classifies the world's economies into four income groups each year on July 1^st^ - high, upper-middle, lower-middle, and low [1]. Lower and Middle Income Countries (LMICs) are currently defined as the 137 countries with the lowest Gross National Income per capita [2].

LMICs already account for 57% of all cancer cases and 65% of deaths globally [3]. The World Health Organisation estimates that 81% of new cancer cases over the next two decades will be diagnosed in an LMIC, many of which are struggling to invest in one of the most complex disease domains to manage [4]. In addition, global cancer research is insufficiently country-centred and too often still highly ‘colonialised’. LMICs have by far the greatest burden of cancer compared with High Income countries (HICs), more complex cancer ecosystems and a paucity of data to inform local cancer policies [5, 6] and support for cancer systems strengthening is abysmal with only 5% of world’s expenditure on cancer in LMIC settings [7].

LMICs have been experiencing increasing cancer-related mortality as a result of rising obesity rates; increasingly sedentary lifestyles; dietary factors; excess tobacco and alcohol use; and persistent carcinogenic infections like Helicobacter pylori, hepatitis B virus, and human papilloma virus, not to mention other contributing factors that are less well understood [8, 9].

ecancer believes that receiving the best possible care is the fundamental human right of every cancer patient across the world. Therefore, there is a clear issue that needs to be addressed in supporting oncology professionals in LMICs through publishing data and providing education on the best clinical practices for their patients and making it accessible to everyone.

The data that informs decision making for cancer management in HICs is often not applicable in LMICs or it needs to be varied to reflect the context. Some LMICs do not have the capacity to perform rigorous clinical trials to assess their own outcomes, so little information currently exists to guide therapeutic management of diagnosed cancers. In addition, there is often little information on whether the cancer drugs they use have adequate performance in populations where the tumour biology, patient-related characteristics, or specific environmental determinants may differ from the HIC populations in which they were initially tested and approved [10]. Another limitation is that pharmaceutical companies drive the majority of the existing clinical research in several world regions. Epidemiological, social, health care systems, independent clinical or implementation research are underfunded due to insufficient governmental or private funding.

To address these issues, *e*cancer commits to giving researchers, healthcare professionals and patients from LMICs a global voice with an opportunity to share clinical experiences and best practice for their patients in their unique environments. *e*cancer also encourages international collaborations which foster an environment of mutually beneficial knowledge sharing and equitable global partnerships. *e*cancer exists to break down any barriers to accessing high quality education by providing it in multiple formats for free as described below.

### ecancer’s Journal (ecancermedicalscience)

In 2017, five times as many research publications were published by the top ten countries in the world than by the bottom 200 [11]. The most common barriers for LMIC researchers to publish in top journals are publication fees, language issues and reviewers criteria for a paper’s acceptance. In order to begin redressing this imbalance, *e*cancer has the following policies for its journal:

- *e*cancer has a ‘pay what you can afford’ model for authors meaning that over 80% of authors publish open access for free and money is never a barrier to publishing vital research
- *e*cancer will only publish research that includes at least one author from a LMIC or the topic directly supports oncology professionals in LMICs or other under-resourced communities
- *e*cancer’s journal is completely open-access which means that all research is freely available to anyone from the moment of publication
- *e*cancer provides native English translations and accepts articles in Spanish
- *e*cancer’s Editorial Board and reviewers comply with a policy that recognises the characteristics, environment and culture of cancer care and global health systems
- *e*cancer believes that good information and research originating in LMICs can also be beneficial to patients and health systems in developed nations in terms of cost reductions, access to care and inequalities for migrants and minorities living in foreign countries.

As a result of these policies, 585 authors from 32 LMICs have published with *e*cancer since 2019 alone.

### ecancer the educational resource

Due to *e*cancer’s focus on supporting oncology professionals from LMICs and the resources that *e*cancer provides to support oncology professionals across the world, a significant number of the learners who visit *e*cancer.org are from these countries. Since the beginning of 2019, 486,475 unique visitors to *e*cancer.org and 11,668 of total registered users are from 137 different LMICs across the world.

*e*cancer attends cancer conferences around the world to interview experts who share experiences of best clinical practice in their region. Due to the fact that *e*cancer is a charity with a different funding model, they are often the only online educational organisation to attend conferences in LMICs and also publish interviews covering topics not covered by anyone else.

As a result, *e*cancer has worked in partnership with organisations such as AORTIC (the African Organisation for Research and Training in Cancer) and BGICC (the Breast- Gynecological & Immuno-oncology International Cancer Conference) to disseminate the latest developments discussed at their conferences.

Since the beginning of 2019, *e*cancer has published 470 interviews with experts based in LMICs to support the dissemination of best clinical practice in their settings.

*e*cancer is also an accredited e-learning provider through the EACCME (European Accreditation Council for Continuing Medical Education). *e*cancer has developed a unique collection of resources focused on the needs of oncology professionals in LMICs including courses on palliative care for those based in Africa, India and Latin America as well as courses focusing on cervical cancer, tobacco dependance and cancer surgery. These courses have been developed in collaboration with organisations such as the African Palliative Care Association, SLACOM (Sociedad Latinoamericana y del Caribe de Oncología Médica), City Cancer Challenge and UICC (the Union for International Cancer Control).

These resources and others have resulted in 2,495 individuals from LMICs taking 7,464 e-learning modules since the beginning of 2019 through *e*cancer. All for free.

Finally, *e*cancer runs educational events in LMICs that focus on choosing wisely and making the best clinical decisions for the patients in the regions and countries that the attendees are from. These events are free or very low cost for attendants and are always run in partnership with local organisations and experts including the National Cancer Grid of India, Liga Colombiana Contra el Cancer and ALCP (Asociación Latinoamericana de Cuidados Paliativos). Historically, these events have been run face to face but have gone virtual during the COVID-19 pandemic including the first patient focused event in Spanish for cancer patients across Latin America.

Since the beginning of 2019, 1,050 learners from LMICs have attended these events with an additional 5,600 virtual attendees.

As an organisation, *e*cancer aims to expand its work in the countries who most need support and do everything it can to address the global imbalance in cancer publishing, education and patient care.

## Conclusion

A huge effort from multiple stakeholders from across the world is needed to combat the current imbalance in global cancer care and outcomes. *e*cancer believes that receiving the best possible care is the fundamental human right of every cancer patient across the world and is therefore aiming to support these efforts. By providing high quality education for free and by supporting authors and researchers from LMICs to publish and access vital research, *e*cancer aims to contribute to the global network of organisations supporting this work.

## Conflicts of interest statement

Danny Burke is the CEO of *e*cancer and has worked for the charity since its formation in 2017.

Prof Richard Sullivan is the Chair of Trustees for the *e*cancer Global Foundation.

Dr Eduardo Cazap is the Editor-in-Chief of *e*cancer’s journal (*e*cancermedicalscience).

## Funding statement

No funding was received for the publication of this article.
